# 4-[(4-Methyl­phen­yl)sulfan­yl]butan-2-one

**DOI:** 10.1107/S1600536813026895

**Published:** 2013-10-09

**Authors:** Sladjana B. Novaković, Zorica Leka, Dragana Stevanović, Jovana Muškinja, Goran A. Bogdanović

**Affiliations:** a Vinča Institute of Nuclear Sciences, Laboratory of Theoretical Physics and Condensed Matter Physics, PO Box 522, University of Belgrade, 11001 Belgrade, Serbia; b Faculty of Metallurgy and Technology, University of Montenegro, Cetinjski put bb, 81000 Podgorica, Montenegro; c Faculty of Sciences, Department of Chemistry, University of Kragujevac, R. Domanovića 12, 34000 Kragujevac, Serbia

## Abstract

In the title compound, C_11_H_14_OS, all non-H atoms are essentially coplanar, with a mean deviation of 0.023 Å. In the crystal, centrosymmetrically related mol­ecules are weakly connected into dimers by pairs of C—H⋯O inter­actions. The dimers are further linked along the *a* axis by weak C—H⋯π and C—H⋯S inter­actions.

## Related literature

For the physico-chemical properties of organosulfur compounds, see: Page (1999 ▶). For the synthetic procedure, see: Stevanović *et al.* (2012 ▶). For the role of sulfur in hydrogen bonding, see: Francuski *et al.* (2011 ▶).
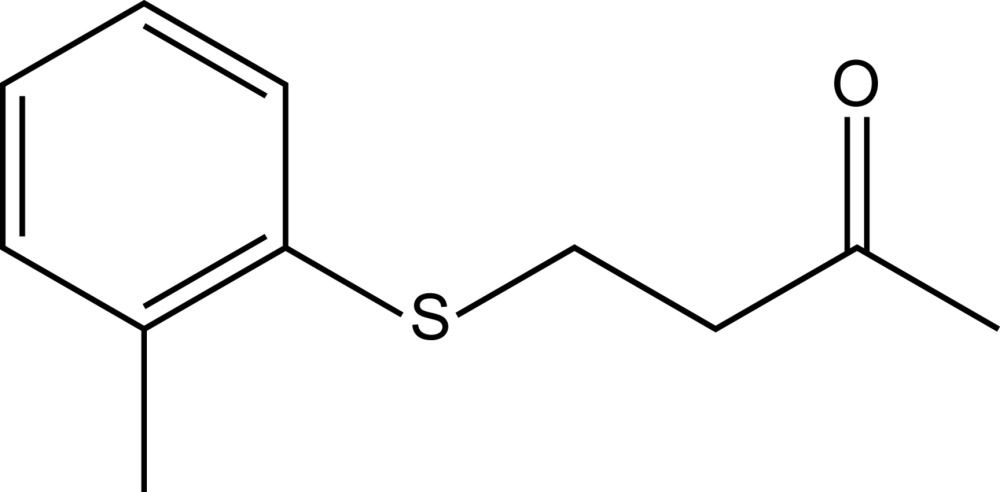


## Experimental

### 

#### Crystal data

C_11_H_14_OS
*M* *_r_* = 194.28
Triclinic, 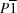

*a* = 7.2703 (11) Å
*b* = 7.3226 (7) Å
*c* = 11.7615 (11) Å
α = 88.232 (8)°
β = 79.343 (10)°
γ = 61.350 (13)°
*V* = 538.80 (13) Å^3^
*Z* = 2
Cu *K*α radiation
μ = 2.33 mm^−1^
*T* = 293 K
0.50 × 0.26 × 0.14 mm

#### Data collection

Agilent Gemini S diffractometer
Absorption correction: multi-scan (*CrysAlis PRO*; Agilent, 2013 ▶) *T*_min_ = 0.444, *T*_max_ = 1.000
3254 measured reflections
2052 independent reflections
1731 reflections with *I* > 2σ(*I*)
*R*_int_ = 0.027

#### Refinement

*R*[*F* ^2^ > 2σ(*F* ^2^)] = 0.055
*wR*(*F* ^2^) = 0.170
*S* = 1.07
2052 reflections
120 parameters
H-atom parameters constrained
Δρ_max_ = 0.34 e Å^−3^
Δρ_min_ = −0.33 e Å^−3^

### 

Data collection: *CrysAlis PRO* (Agilent, 2013 ▶); cell refinement: *CrysAlis PRO*; data reduction: *CrysAlis PRO*; program(s) used to solve structure: *SHELXS97* (Sheldrick, 2008 ▶); program(s) used to refine structure: *SHELXL97* (Sheldrick, 2008 ▶); molecular graphics: *ORTEP-3* (Farrugia, 2012 ▶) and *Mercury* (Macrae *et al.*, 2006 ▶); software used to prepare material for publication: *WinGX* (Farrugia, 2012 ▶), *PLATON* (Spek, 2009 ▶) and *PARST* (Nardelli, 1995 ▶).

## Supplementary Material

Crystal structure: contains datablock(s) I, global. DOI: 10.1107/S1600536813026895/rz5084sup1.cif

Structure factors: contains datablock(s) I. DOI: 10.1107/S1600536813026895/rz5084Isup2.hkl

Supplementary material file. DOI: 10.1107/S1600536813026895/rz5084Isup3.cml

Additional supplementary materials:  crystallographic information; 3D view; checkCIF report

## Figures and Tables

**Table 1:** Hydrogen-bond geometry (Å, °) *Cg* is the centroid of the C4–C9 phenyl ring.

*D*—H⋯*A*	*D*—H	H⋯*A*	*D*⋯*A*	*D*—H⋯*A*
C10—H10c⋯O1^i^	0.96	2.67	3.579 (4)	158
C3—H3a⋯S1^ii^	0.97	2.99	3.855 (3)	149
C3—H3b⋯S1^iii^	0.97	3.02	3.870 (3)	147
C2—H2a⋯*Cg* ^ii^	0.97	2.86	3.628 (4)	137
C2—H2b⋯*Cg* ^iv^	0.97	2.95	3.678 (4)	133

Symmetry codes: (i) 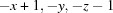; (ii) 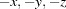; (iii) 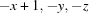; (iv) 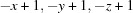.

**Fig. 1.**
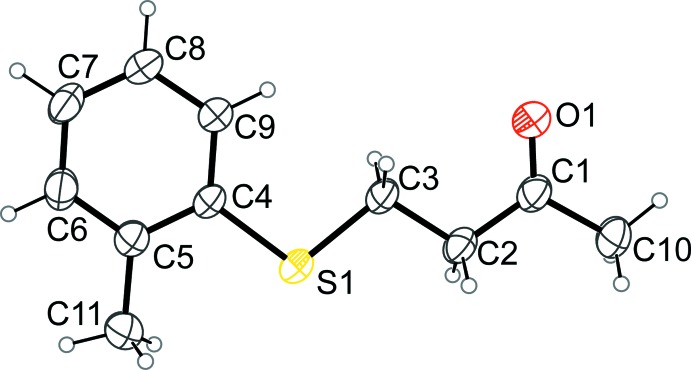
The molecular structure of the title compound, with atom labels and 40% probability displacement ellipsoids for non-H atoms.

**Fig. 2.**
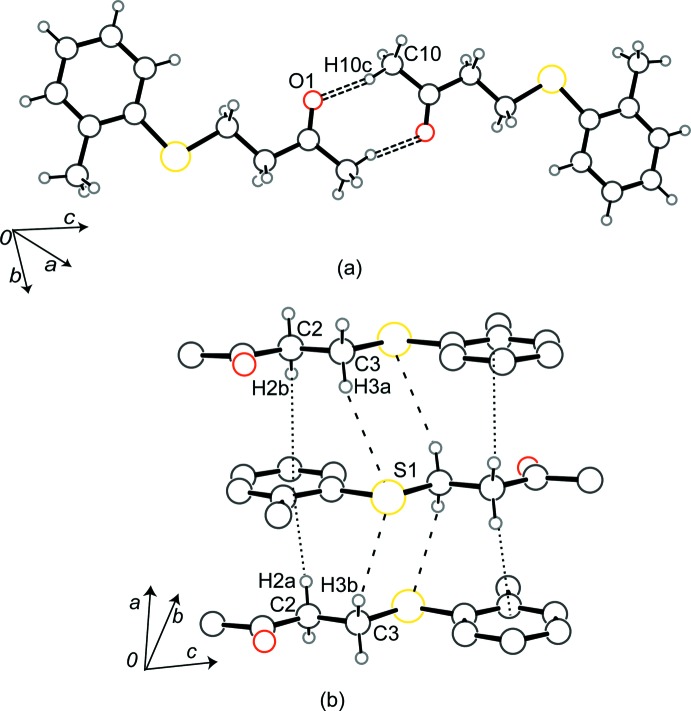
Intermolecular interactions in the title compound: (a) C—H···O interactions (dashed lines) connecting centrosymmetrically related molecules into dimers; (b) C—H···π (dotted lines) and C—H···S interactions (dashed lines) connecting the molecules along a axis. H-atoms not involved in hydrogen interactions are omitted.

## supplementary crystallographic information

### 1. Comment

Sulfides containing a carbonyl group are versatile precursors for the synthesis of wide range of biologically interesting compounds (Page, 1999). The main approach to β-thiaketones is the addition of compounds containing an SH group to conjugated carbonyls (the thia-Michael reaction). We recently published a versatile method for electrochemical generation of the catalyst for this addition (Stevanović *et al.*, 2012) and herein we report the structure of 4-(*o*-tolylthio)butan-2-one.

The molecule of the title compound (Fig. 1) is essentially planar with a mean deviation of all non-H atoms of 0.023 Å. Atoms O1 and C10 exhibit the highest deviation from the mean molecular plane of 0.047 (3) and -0.057 (2) Å, respectively. The crystal packing displays no classical hydrogen bonding. The carbonyl O1 acceptor is engaged only in a weak C10—H10*c*···O1 interaction (Table 1) which associates the centrosymmetric molecules into dimers (Fig. 2a). Pairs of C—H···π and C—H···S interactions (Table 1) connect the molecules along the *a* axis (Figure 2b). In the absence of more relevant hydrogen bonding the weak C—H···S interactions can be considered important for the stabilization of the crystal structure (Francuski *et al.*, 2011).

### 2. Experimental

The title compound was obtained by treating methyl vinyl ketone with the corresponding thiophenol in the presence of an electrochemically generated zirconium catalyst, following the reported procedure (Stevanović *et al.*, 2012).

### 3. Refinement

All H atoms were placed at geometrically calculated positions and included in the refinement in the riding model approximation, with C—H lengths of 0.93 (CH), 0.96 (CH_3_) and 0.97 (CH_2_) Å. *U*_iso_ of the H atoms was set at 1.5*U*_eq_ of the parent C atom for the methyl group and at 1.2*U*_eq_ otherwise.

### Crystal data

**Table:**

C_11_H_14_OS	*Z* = 2
*M**_r_* = 194.28	*F*(000) = 208
Triclinic, *P*1	*D*_x_ = 1.198 Mg m^−^^3^
Hall symbol: -P 1	Cu *K*α radiation, λ = 1.54180 Å
*a* = 7.2703 (11) Å	Cell parameters from 1309 reflections
*b* = 7.3226 (7) Å	θ = 7.0–72.1°
*c* = 11.7615 (11) Å	µ = 2.33 mm^−^^1^
α = 88.232 (8)°	*T* = 293 K
β = 79.343 (10)°	Prismatic, colourless
γ = 61.350 (13)°	0.50 × 0.26 × 0.14 mm
*V* = 538.80 (13) Å^3^	

### Data collection

**Table:**

Agilent Gemini S diffractometer	2052 independent reflections
Radiation source: Enhance (Cu) X-ray Source	1731 reflections with *I* > 2σ(*I*)
Graphite monochromator	*R*_int_ = 0.027
Detector resolution: 16.3280 pixels mm^-1^	θ_max_ = 72.9°, θ_min_ = 3.8°
ω scans	*h* = −8→8
Absorption correction: multi-scan (*CrysAlis PRO*; Agilent, 2013)	*k* = −9→5
*T*_min_ = 0.444, *T*_max_ = 1.000	*l* = −14→14
3254 measured reflections	

### Refinement

**Table:**

Refinement on *F*^2^	Primary atom site location: structure-invariant direct methods
Least-squares matrix: full	Secondary atom site location: difference Fourier map
*R*[*F*^2^ > 2σ(*F*^2^)] = 0.055	Hydrogen site location: inferred from neighbouring sites
*wR*(*F*^2^) = 0.170	H-atom parameters constrained
*S* = 1.07	*w* = 1/[σ^2^(*F*_o_^2^) + (0.1046*P*)^2^ + 0.083*P*] where *P* = (*F*_o_^2^ + 2*F*_c_^2^)/3
2052 reflections	(Δ/σ)_max_ < 0.001
120 parameters	Δρ_max_ = 0.34 e Å^−^^3^
0 restraints	Δρ_min_ = −0.33 e Å^−^^3^

### Fractional atomic coordinates and isotropic or equivalent isotropic displacement parameters (Å^2^)

**Table:**

	*x*	*y*	*z*	*U*_iso_*/*U*_eq_	
S1	0.31244 (10)	−0.14778 (8)	0.03374 (5)	0.0640 (3)	
O1	0.2948 (4)	0.0791 (3)	−0.32862 (19)	0.0927 (7)	
C1	0.3882 (4)	−0.1053 (4)	−0.3166 (2)	0.0643 (6)	
C2	0.4007 (4)	−0.1919 (4)	−0.2004 (2)	0.0596 (6)	
H2A	0.5496	−0.2765	−0.1949	0.072*	
H2B	0.3359	−0.2818	−0.1924	0.072*	
C3	0.2892 (4)	−0.0231 (4)	−0.1015 (2)	0.0576 (6)	
H3A	0.1398	0.0627	−0.1058	0.069*	
H3B	0.3552	0.0656	−0.1070	0.069*	
C4	0.1796 (3)	0.0637 (3)	0.14013 (19)	0.0524 (5)	
C5	0.1709 (4)	0.0158 (4)	0.2556 (2)	0.0598 (6)	
C6	0.0713 (5)	0.1782 (4)	0.3408 (2)	0.0709 (7)	
H6	0.0662	0.1474	0.4182	0.085*	
C7	−0.0202 (4)	0.3834 (4)	0.3140 (2)	0.0714 (7)	
H7	−0.0864	0.4896	0.3728	0.086*	
C8	−0.0132 (4)	0.4303 (4)	0.2004 (2)	0.0687 (7)	
H8	−0.0762	0.5690	0.1819	0.082*	
C9	0.0874 (4)	0.2723 (4)	0.1125 (2)	0.0639 (6)	
H9	0.0935	0.3050	0.0353	0.077*	
C10	0.4997 (5)	−0.2615 (5)	−0.4198 (2)	0.0793 (8)	
H10A	0.4179	−0.3296	−0.4283	0.119*	
H10B	0.6391	−0.3635	−0.4083	0.119*	
H10C	0.5132	−0.1911	−0.4884	0.119*	
C11	0.2678 (6)	−0.2074 (4)	0.2878 (3)	0.0811 (8)	
H11A	0.2357	−0.2111	0.3705	0.122*	
H11B	0.4198	−0.2749	0.2614	0.122*	
H11C	0.2095	−0.2787	0.2519	0.122*	

### Atomic displacement parameters (Å^2^)

**Table:**

	*U*^11^	*U*^22^	*U*^33^	*U*^12^	*U*^13^	*U*^23^
S1	0.0807 (5)	0.0453 (4)	0.0554 (4)	−0.0233 (3)	−0.0076 (3)	−0.0088 (2)
O1	0.1279 (18)	0.0579 (11)	0.0675 (12)	−0.0307 (12)	−0.0024 (11)	−0.0022 (9)
C1	0.0754 (15)	0.0555 (13)	0.0581 (13)	−0.0312 (12)	−0.0025 (11)	−0.0110 (10)
C2	0.0651 (14)	0.0516 (12)	0.0570 (13)	−0.0263 (11)	−0.0029 (10)	−0.0126 (10)
C3	0.0650 (13)	0.0497 (12)	0.0524 (12)	−0.0248 (10)	−0.0043 (10)	−0.0106 (9)
C4	0.0569 (12)	0.0455 (11)	0.0511 (11)	−0.0225 (9)	−0.0066 (9)	−0.0084 (8)
C5	0.0675 (14)	0.0561 (13)	0.0554 (12)	−0.0299 (11)	−0.0101 (10)	−0.0004 (10)
C6	0.0868 (18)	0.0749 (17)	0.0496 (13)	−0.0406 (14)	−0.0041 (12)	−0.0053 (11)
C7	0.0769 (16)	0.0612 (15)	0.0637 (15)	−0.0276 (13)	0.0019 (12)	−0.0204 (12)
C8	0.0773 (16)	0.0463 (12)	0.0700 (15)	−0.0221 (11)	−0.0061 (12)	−0.0092 (11)
C9	0.0798 (16)	0.0493 (12)	0.0549 (13)	−0.0258 (11)	−0.0104 (11)	−0.0016 (10)
C10	0.106 (2)	0.0705 (17)	0.0544 (14)	−0.0401 (16)	−0.0038 (14)	−0.0132 (12)
C11	0.113 (2)	0.0628 (16)	0.0651 (16)	−0.0389 (16)	−0.0217 (15)	0.0098 (12)

### Geometric parameters (Å, º)

**Table:**

S1—C4	1.771 (2)	C6—C7	1.375 (4)
S1—C3	1.804 (2)	C6—H6	0.9300
O1—C1	1.204 (3)	C7—C8	1.367 (4)
C1—C2	1.488 (4)	C7—H7	0.9300
C1—C10	1.506 (3)	C8—C9	1.388 (3)
C2—C3	1.522 (3)	C8—H8	0.9300
C2—H2A	0.9700	C9—H9	0.9300
C2—H2B	0.9700	C10—H10A	0.9600
C3—H3A	0.9700	C10—H10B	0.9600
C3—H3B	0.9700	C10—H10C	0.9600
C4—C5	1.390 (3)	C11—H11A	0.9600
C4—C9	1.398 (3)	C11—H11B	0.9600
C5—C6	1.386 (3)	C11—H11C	0.9600
C5—C11	1.506 (3)		
			
C4—S1—C3	103.72 (11)	C7—C6—H6	119.1
O1—C1—C2	122.4 (2)	C5—C6—H6	119.1
O1—C1—C10	121.3 (3)	C8—C7—C6	119.5 (2)
C2—C1—C10	116.4 (2)	C8—C7—H7	120.2
C1—C2—C3	112.78 (19)	C6—C7—H7	120.2
C1—C2—H2A	109.0	C7—C8—C9	120.4 (2)
C3—C2—H2A	109.0	C7—C8—H8	119.8
C1—C2—H2B	109.0	C9—C8—H8	119.8
C3—C2—H2B	109.0	C8—C9—C4	119.9 (2)
H2A—C2—H2B	107.8	C8—C9—H9	120.1
C2—C3—S1	108.38 (16)	C4—C9—H9	120.1
C2—C3—H3A	110.0	C1—C10—H10A	109.5
S1—C3—H3A	110.0	C1—C10—H10B	109.5
C2—C3—H3B	110.0	H10A—C10—H10B	109.5
S1—C3—H3B	110.0	C1—C10—H10C	109.5
H3A—C3—H3B	108.4	H10A—C10—H10C	109.5
C5—C4—C9	119.8 (2)	H10B—C10—H10C	109.5
C5—C4—S1	117.25 (18)	C5—C11—H11A	109.5
C9—C4—S1	122.96 (18)	C5—C11—H11B	109.5
C6—C5—C4	118.5 (2)	H11A—C11—H11B	109.5
C6—C5—C11	120.6 (2)	C5—C11—H11C	109.5
C4—C5—C11	120.9 (2)	H11A—C11—H11C	109.5
C7—C6—C5	121.9 (2)	H11B—C11—H11C	109.5

### Hydrogen-bond geometry (Å, º)

Cg is the centroid of the C4–C9 phenyl ring.

**Table:**

*D*—H···*A*	*D*—H	H···*A*	*D*···*A*	*D*—H···*A*
C10—H10c···O1^i^	0.96	2.67	3.579 (4)	158
C3—H3a···S1^ii^	0.97	2.99	3.855 (3)	149
C3—H3b···S1^iii^	0.97	3.02	3.870 (3)	147
C2—H2a···*Cg*^ii^	0.97	2.86	3.628 (4)	137
C2—H2b···*Cg*^iv^	0.97	2.95	3.678 (4)	133

Symmetry codes: (i) −*x*+1, −*y*, −*z*−1; (ii) −*x*, −*y*, −*z*; (iii) −*x*+1, −*y*, −*z*; (iv) −*x*+1, −*y*+1, −*z*+1.
